# Intramedullary humeral replacement: an evolving design

**DOI:** 10.1051/sicotj/2015045

**Published:** 2016-02-02

**Authors:** Ali Abdullah Mohammed, Simon Peter Frostick

**Affiliations:** 1 Royal Liverpool University Hospital Liverpool L69 3GA UK

**Keywords:** Humeral replacement, Shoulder replacement, Elbow replacement, Megaprosthesis, Endo-prosthetic replacement, Humeral replacement, Shoulder replacement, Elbow replacement, Megaprosthesis, Endo-prosthetic replacement

## Abstract

*Introduction*: Total humeral replacement is used to reconstruct the upper limb after tumour resection, while in cases of complex revisions for non-oncological reasons, using tumour prosthesis implants will lead to an otherwise avoidable further bone resection and violation of the surrounding tissues. This report describes a design evolution in three non-oncological cases, where a total humeral resection to perform a total humeral replacement is avoided and instead the simultaneous shoulder and elbow replacements were connected via custom-made intramedullary linkages.

*Methods*: Three cases of simultaneous shoulder and elbow replacement were performed for complex revision situations over a period of 42 months. They were performed while preserving as much humeral bone stock as possible, with the design changing from a big intramedullary connecting stem to a smaller component when performing an Intramedullary Humeral Replacement (IMHR), allowing preservation of more bone and soft tissue attachment than if a total humeral replacement were performed.

*Results*: None had any neurovascular complication or any further revision for the humeral replacement, or the shoulder and elbow components.

*Discussion*: We have showed three examples of an evolving design aiming to preserve as much of the anatomy as possible to help in decreasing the surgical impact and invasiveness of this procedure, while doing less bone resection and sacrificing less of the soft tissue attachments.

## Introduction

Total humeral replacement with simultaneous shoulder and elbow replacement (SSER) is an uncommon but known surgical procedure. Most cases which need humeral replacement were reported in the literature and underwent reconstructive post-tumor resection procedures. However, a similar procedure is also an option for complicated revision situations that result after repeated revisions for shoulder and elbow replacements in non-oncological situations, where a total humeral replacement would be needed to connect the revision shoulder and elbow replacement components.

There is lack of consensus on the most reliable design to address these complex situations. However to our best knowledge, this is the first report to describe “bypassing” the humeral shaft rather than resecting and replacing it when performing a SSER for complex revision cases where the humeral bone stock is not great to start with. So instead of adding insult to injury by resecting even more humeral bone, it is more practical and less invasive to preserve the remaining humeral bone and hence preserve some of the soft tissue attachment. This helps in avoiding the violation of its soft tissue envelope aiming for less complication rate, making the procedure technically easier than if the SSER included resection of the humerus, or the remaining part, and replacing it with a sizeable bulky metallic humerus.

In this report, we will illustrate three cases where the humerus although did not have enough bone stock to perform ipsilateral shoulder and elbow replacement while maintaining enough bony support for the prosthesis. Still the need for total humeral replacement was substituted with intramedullary rod connecting the shoulder and the elbow components, while maintaining the humeral bony shell to still provide undisturbed site for muscular and soft tissue attachments.

## Materials and methods

Three complex revision shoulder and elbow arthroplasty cases needed limb reconstruction, which were performed using custom-made but modular Biomet SSER implants.

All the three cases were revision cases. They had the new SSER prosthesis as a patient-specific custom-made implant with IMHR, prior to which they had multiple operations which left them with deficient humeral bone stock, with the case-specific details being as follows:

The first case: Post polytrauma due to car collision, after which he needed multiple operations including shoulder and elbow replacements that had to be revised, which left him with significant humeral bone loss.

The second case: Had shoulder replacement and revision elbow replacement, after which the patient had trauma and developed a peri-prosthetic fracture, which progressed into aseptic loosening within a deficient humeral envelope.

The third case: Revision total elbow arthroplasty for aseptic loosening (primary was 15 years prior to revision), re-revised after 5 years, ended with fixed flexion deformity of the elbow. Also had right shoulder hemi-arthroplasty (which has survived over 17 years); however, the hemi-arthroplasty stem stress shielded the outer cortex of the upper humerus.

**Figure 1.**
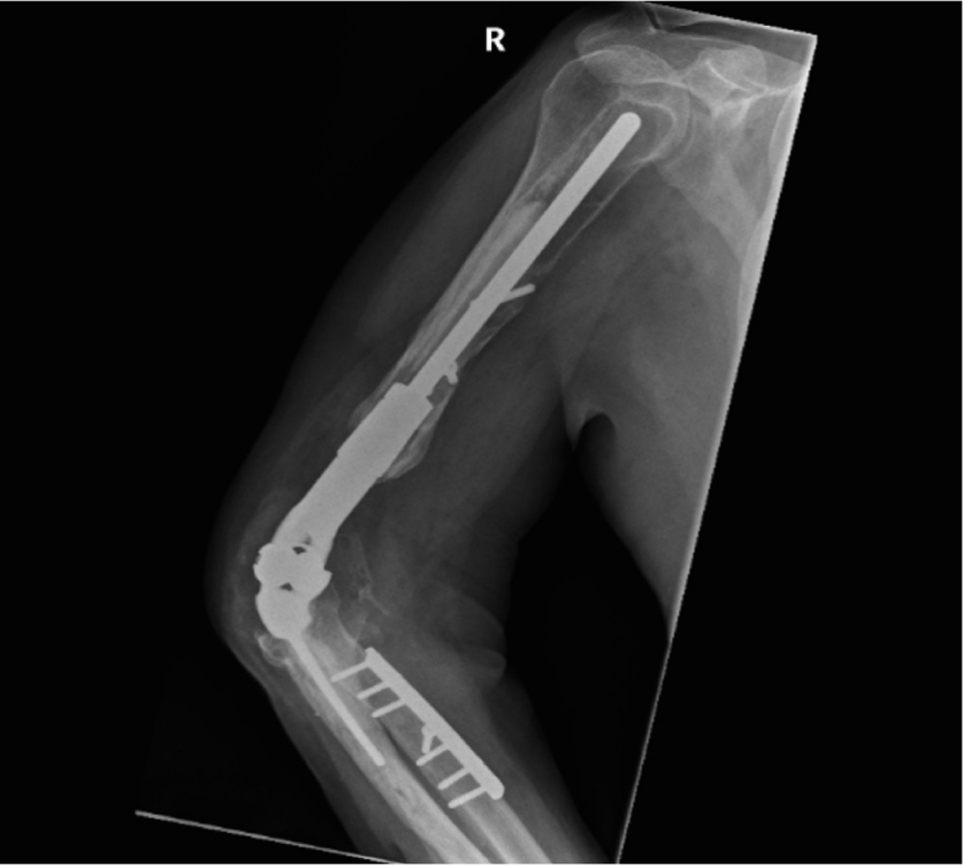
This figure shows the pre-operative radiograph for the first case, demonstrating a complex situation with significant loosening around the stem of a previous endo-prosthetic replacement, with a poor bony envelope remaining.

**Figure 2.**
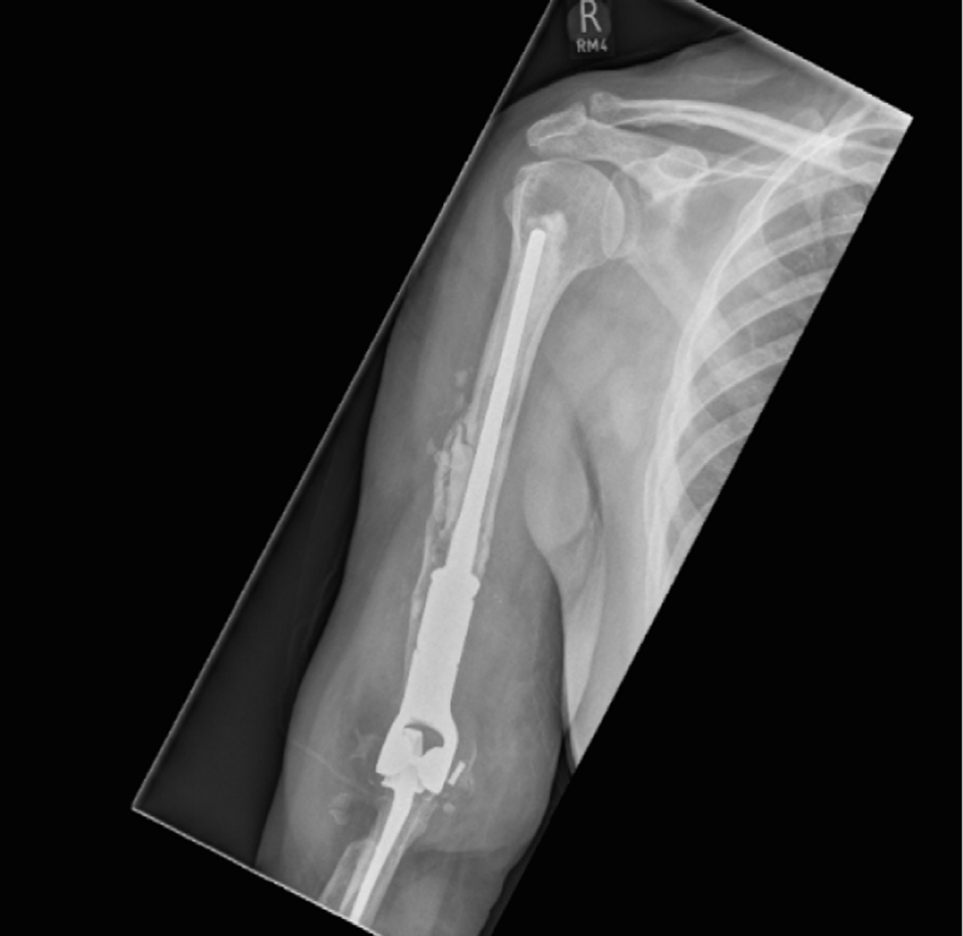
This figure shows the pre-operative radiograph for the second case, which also shows significant loosening around the endo-prosthetic stem with a peri-prosthetic fracture and a broken elbow prosthetic hinge.

**Figure 3.**
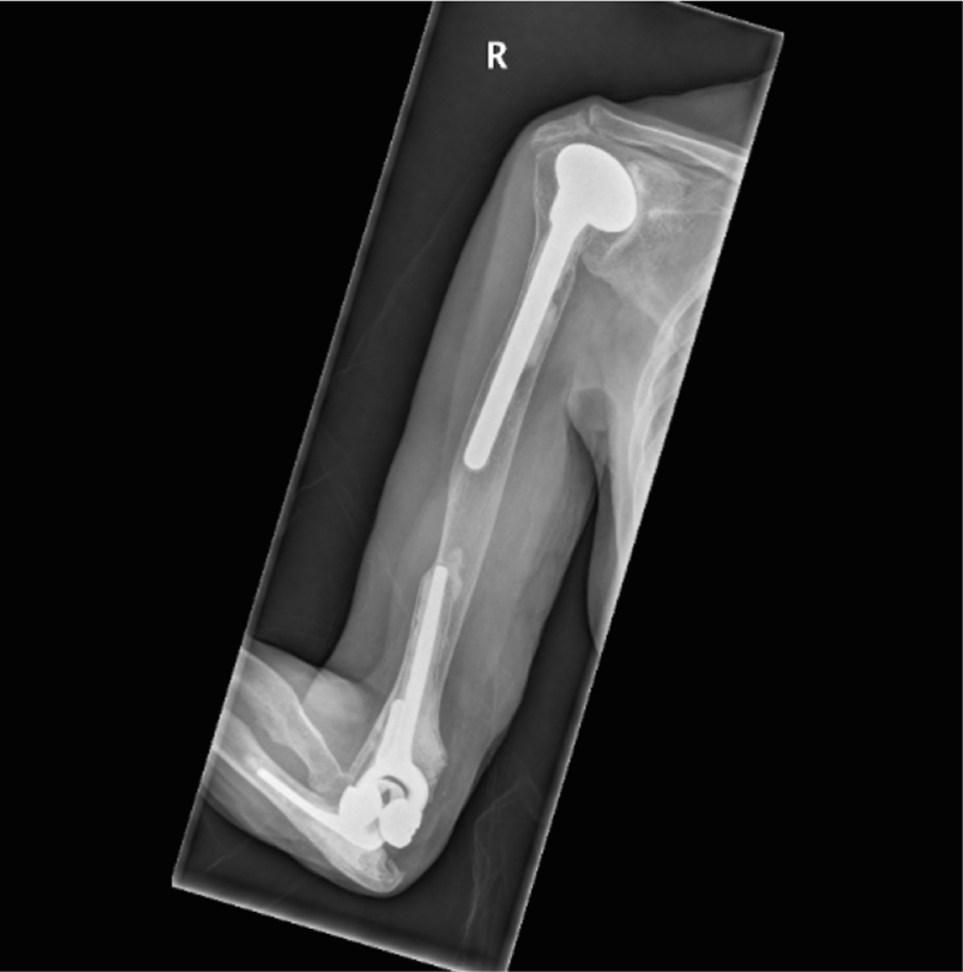
This figure shows the pre-operative radiograph for the third case, demonstrating an elbow replacement and a shoulder hemi-arthroplasty on the same side, with poor bone quality, loosening, and impending fracture.

**Figure 4.**
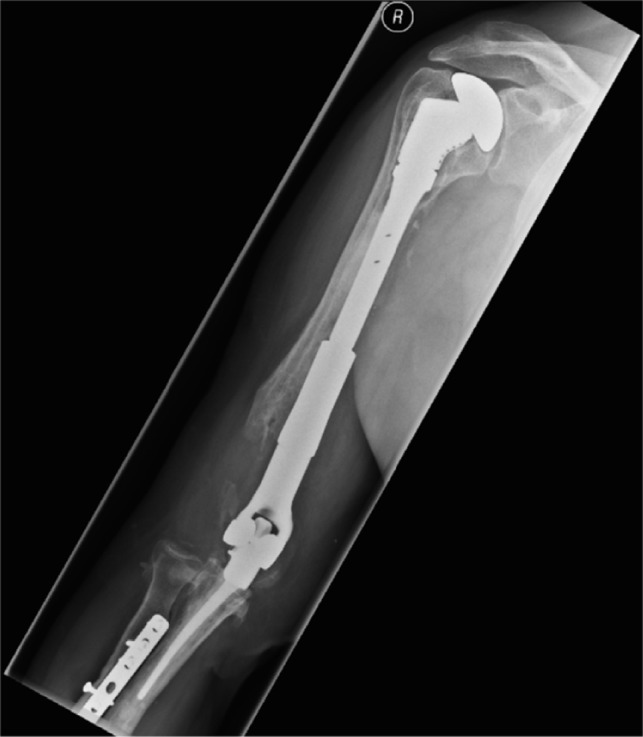
The radiograph shows IMHR for the first case, and it illustrates preservation of mainly the proximal third of the humerus, with the usage of shoulder hemi-arthroplasty proximally and a large humeral body distally joined to an elbow replacement ulnar component.

**Figure 5.**
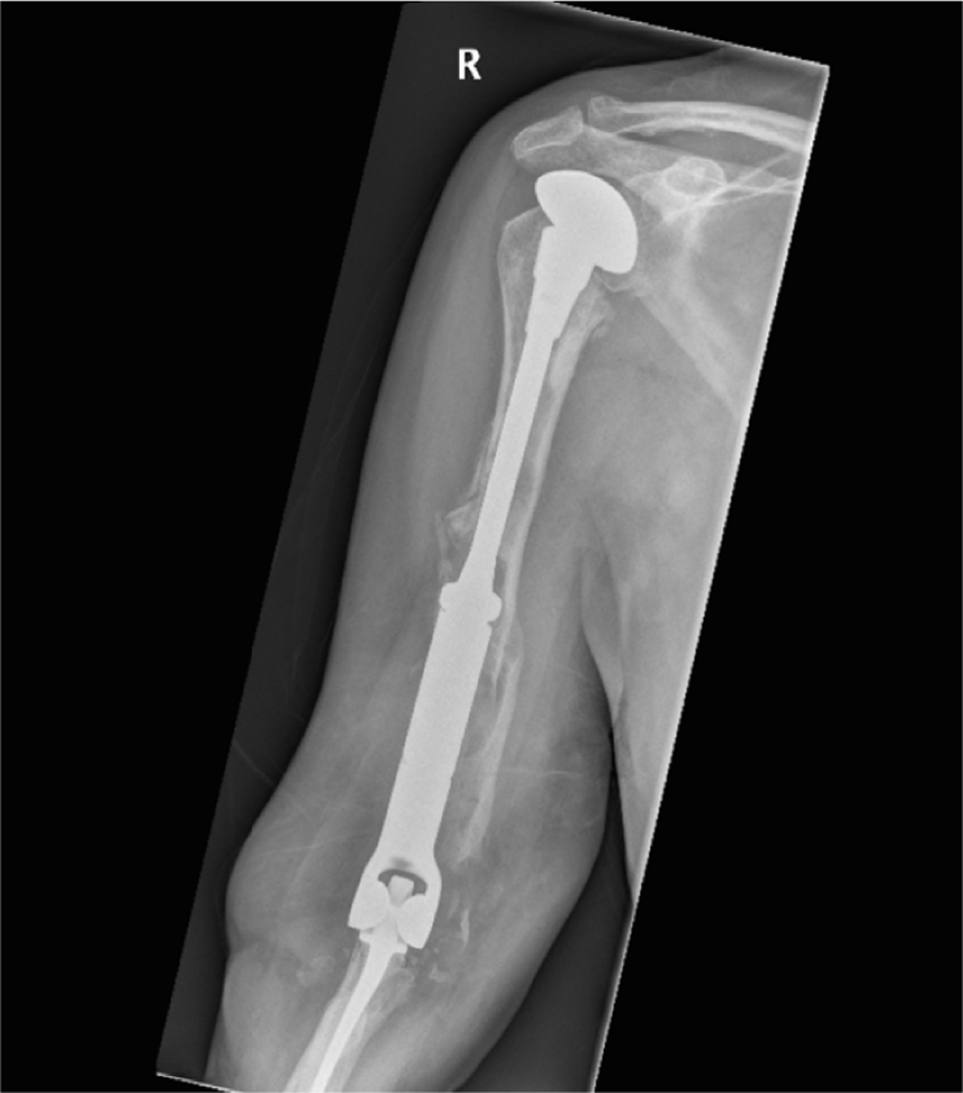
The radiograph shows IMHR for the second case, and it illustrates preservation of most of the proximal half of the humeral shaft with a shoulder hemi-arthroplasty proximally and a large humeral body distally joined to an elbow replacement ulnar component.

**Figure 6.**
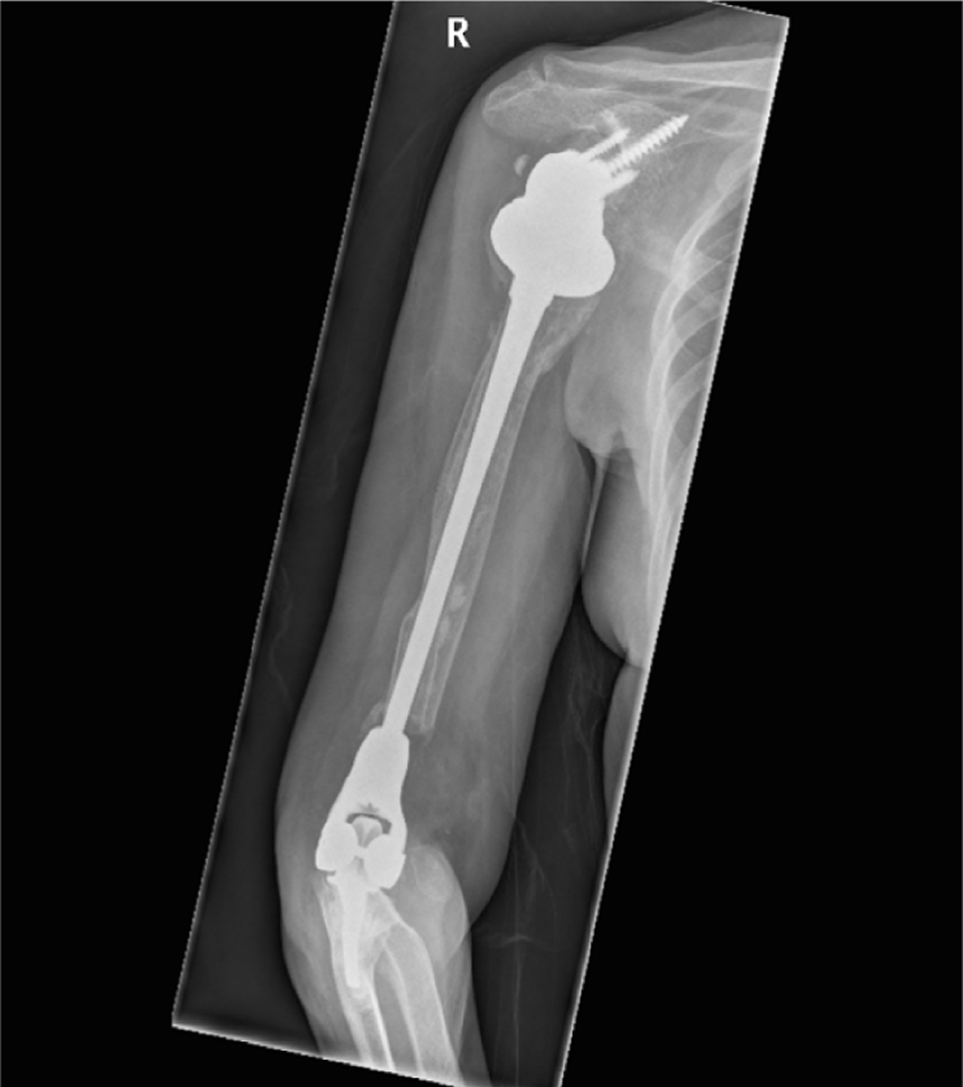
The radiograph shows IMHR for the third case, and it illustrates preservation of most of the humeral shaft, which is bypassed by an intramedullary rod, while using a reverse shoulder replacement component proximally and a smaller distal humeral prosthetic body over an elbow replacement ulnar component.

In Figures 1–3 the pre-operative radiographs are illustrated, while in Figures 4–6, the most recent post IMHR X-rays are shown to demonstrate the design progression.

**Figure 7.**
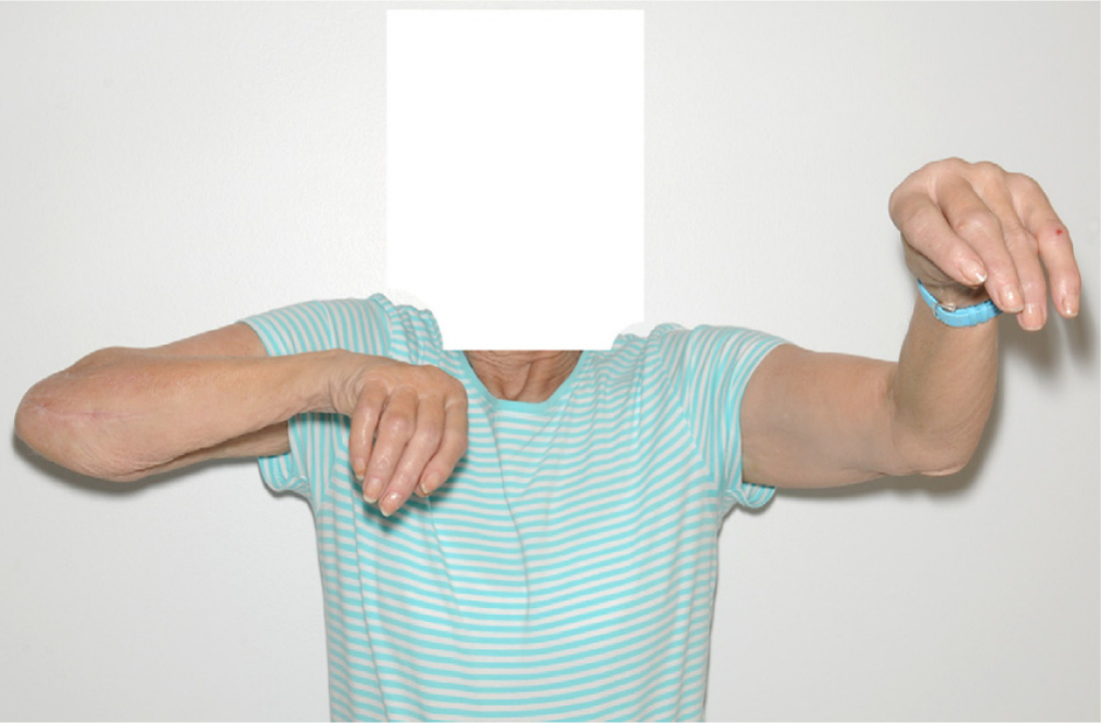
A front view clinical photograph for the third case showing active shoulder lateral abduction.

**Figure 8.**
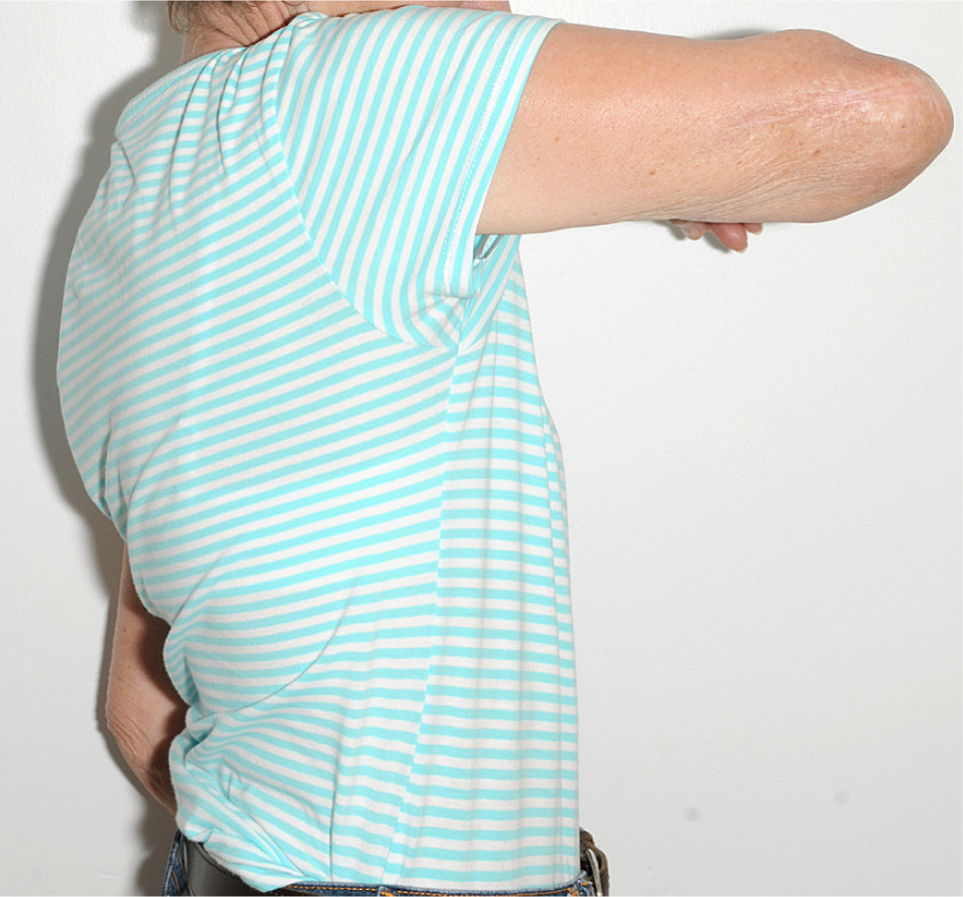
A side view clinical photograph for the third case showing active shoulder forward flexion.

**Figure 9.**
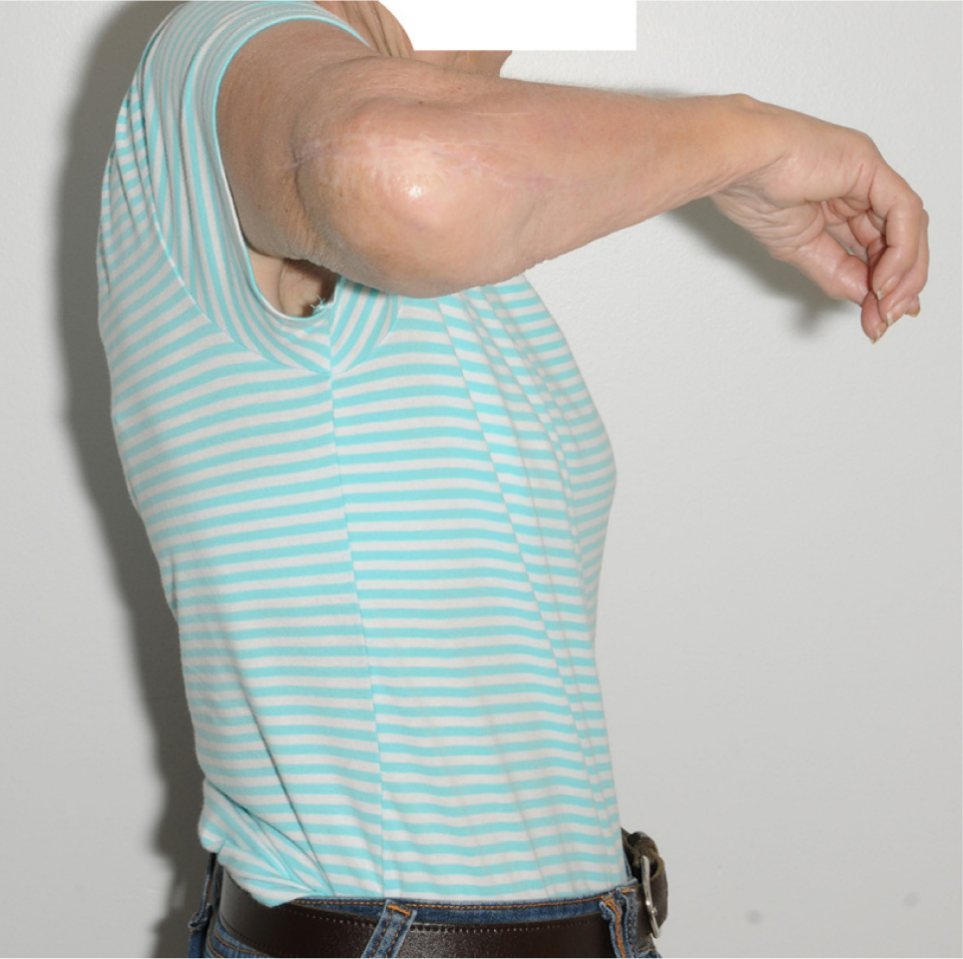
A side view clinical photograph of the third case showing active shoulder lateral abduction.

In Figures 7–9, post-operative clinical photographs for the third case are illustrated.

### Surgical technique

The procedure is done under general anaesthesia in beach chair position, with prophylactic antibiotics at induction.

The surgical approach has three major components:

1. A delto-pectoral approach is needed to replace the shoulder and expose the proximal edge of the humeral shaft.
2. A posterior approach of the elbow, while the arm is kept across the patient’s chest, this exposure is used to replace the elbow and to expose the distal aspect of the humeral shaft.
3. An intramedullary rod is passed through the humeral canal without dissecting around the humeral shaft.

This rod connects the shoulder and the elbow replacements.

## Results

None had any neurovascular complication or any further revision for the IMHR, and follow-up post-operative outcome scorings are as follows:

First case at 24 months post-operatively: DASH 40.9, SF12 of 42.5 on the physical component & SF12 of 56.6 on the mental component.
Second case at 6 months post-operatively: DASH 56.8, SF12 of 26.47 on the physical component & SF12 of 68.88 on the mental component.
Third case at 7 months post-operatively: DASH 63.6, SF12 of 26.1 on the physical component & SF12 of 62.4 on the mental component.

## Discussion

Total Humeral Endoprosthetic Replacement (THER) has been reported in the literature in post-malignant tumour resection, and described as safe, consistent with predictable results, and low rates of complication [1]. It has especially been possible, from oncological prospective, to perform limb salvage procedures with the use of effective chemotherapy without compromising long-term survival [2].

Post-tumour resection megaprosthesis designs have evolved with time from custom-made mono-block to more modular designs [3].

THER 90% 10-year survivorship is previously reported [4]; however, most reports in the literature seem to address post-tumour resection upper limb reconstruction, making megaprosthesis an available option, which has extended its usage in non-oncological situations, a procedure that would need further bone resection to fit the prosthesis in place [3].

Doing complex revisions for non-oncological reasons with SSER, while using a tumour prosthesis can remove more bone, further scarifies tissue attachment than actually necessary, and needs larger exposure, which would impose further risk on the neurovascular structures that surround the remaining humeral shaft bony envelope.

An IMHR would only be meaningful if some of the humerus is still preserved and hence worth saving from further resection.

In this report, the three cases described did show design progression to an intramedullary “bypass” humeral replacement, which was technically easier to perform by linking the shoulder and the elbow components via a less bulky humeral stem, which “bypassed” the remaining humerus without violating its surrounding structures. They also showed no early complications and good long-term outcome for the first case.

## Conflict of interest

AAM declares no conflict of interest in relation with this paper. SPF is a consultant for Biomet and co-designer of the implant.
